# Mapping centromeres of microchromosomes in the zebra finch (*Taeniopygia guttata*) using half-tetrad analysis

**DOI:** 10.1007/s00412-015-0560-7

**Published:** 2015-12-15

**Authors:** Ulrich Knief, Wolfgang Forstmeier

**Affiliations:** Department of Behavioural Ecology and Evolutionary Genetics, Max Planck Institute for Ornithology, 82319 Seewiesen, Germany

**Keywords:** Linkage analysis, Half-tetrad, Chromosomal anomalies, Telomere, Polyploidy

## Abstract

Centromeres usually consist of hundreds of kilobases of repetitive sequence which renders them difficult to assemble. As a consequence, centromeres are often missing from assembled genomes and their locations on physical chromosome maps have to be inferred from flanking sequences via fluorescence in situ hybridization (FISH). Alternatively, centromere positions can be mapped using linkage analyses in accidentally triploid individuals formed by half-tetrads (resulting from the inheritance of two chromatids from a single meiosis). The current genome assembly of the zebra finch (*Taeniopygia guttata*) comprises 32 chromosomes, but only for the ten largest chromosomes centromere positions have been mapped using FISH. We here map the positions of most of the remaining centromeres using half-tetrad analyses. For this purpose, we genotyped 37 zebra finches that were triploid or tetraploid due to inheritance errors (and mostly died as embryos) together with their parents at 64 microsatellite markers (at least two per chromosome). Using the information on centromere positions on the ten largest chromosomes, we were able to identify 12 cases of non-disjunction in maternal meiosis I and 10 cases of non-disjunction in maternal meiosis II. These 22 informative cases allowed us to infer centromere positions on additional 19 microchromosomes in reference to the current genome assembly. This knowledge will be valuable for studies of chromosome evolution, meiotic drive and species divergence in the avian lineage.

**Electronic supplementary material:**

The online version of this article (doi:10.1007/s00412-015-0560-7) contains supplementary material, which is available to authorized users.

## Introduction

Centromeres are the attachment sites of the spindle microtubules and are essential for the proper segregation of chromosomes in mitosis and meiosis. The location of centromeres can be readily identified by means of cytogenetic methods, yet integrating the cytogenetic with the linkage/physical map can be quite difficult, because centromeres are not defined by a specific sequence motive; they usually consist of hundreds of kilobases of repetitive sequence and because of that, they are often missing from assembled genomes (Krasikova et al. 2006; Shang et al. 2010). However, it has been recently suggested that centromeric/telomeric DNA prominently contributes to species divergence in birds and mammals (Carneiro et al. 2009; Ellegren et al. 2012) and because of that, more knowledge about the position of these cytogenetic features is needed for any reference genome.

The zebra finch (*Taeniopygia guttata*) was the second avian species whose genome was sequenced, and it was assembled into 32 chromosomes using bacterial artificial chromosomes (BACs) and a linkage map (Warren et al. 2010). With its genomic resources at hand, it is arguably qualitatively the second best annotated avian genome after the chicken (*Gallus gallus*), even though karyotypically the genome consists of n = 40 chromosomes (Pigozzi and Solari 1998), meaning that eight chromosomes have not been assembled yet. Throughout this paper we will use the chromosome nomenclature introduced by Itoh and Arnold (2005) and Warren et al. (2010).

Bird genomes consist of a few large macro- and several smaller microchromosomes; the exact definition of them being rather loose. It is generally accepted that the zebra finch genome consists of seven macrochromosomes (*Tgu1*–*Tgu5*, *Tgu1A* and *TguZ*; Itoh and Arnold 2005) with an assembled size range of 62–156 Mb and 33 microchromosomes ranging from 9 kb to 40 Mb.

Notwithstanding the amount of genomic and molecular tools available for the zebra finch, only for the ten largest of the 32 assembled chromosomes the location of the centromere is known in reference to the physical map (WUSTL v3.2.4; Warren et al. 2010). These positions were inferred from flanking sequences via fluorescence in situ hybridization (FISH). Among those ten chromosomes, seven are submetacentric (*Tgu1*, *Tgu1A*, *Tgu2*, *Tgu3*, *Tgu4*, *Tgu7*, *TguZ*), meaning that the centromere is located slightly off the middle of the chromosome, and the remaining more or less acrocentric (*Tgu5*, *Tgu6*, *Tgu8*), meaning that the centromere is located on either end of the chromosome (Pigozzi and Solari 1998). Chromosome *Tgu5*, which is the sixth largest chromosome in the zebra finch karyotype, is known to be polymorphic for a pericentric inversion which changes the chromosome to be submetacentric (Christidis 1986; Itoh and Arnold 2005). All other chromosomes that are smaller than chromosome *Tgu8*, including those that are not yet assembled, are known to be acrocentric from cytogenetic studies (Pigozzi 2008). However, their centromeric ends have not been distinguished from their distal ends in reference to the physical map.

To fill this gap, we here apply centromere-marker-mapping using linkage analysis in half-tetrads, which requires that at least two chromatids of a single meiosis are recovered together (Mather 1938). In a normal meiosis, homologous chromosomes are separated in the first meiotic division (meiosis I) and sister chromatids in the second meiotic division (meiosis II), giving rise to four haploid gametes. In the female meiosis, three of these haploid cells degenerate (the so called polar bodies) and a single oocyte survives. Centromeres are the attachment sites for the spindle microtubules that mediate the separation of chromosomes in meiosis I and II. Accordingly, in the first meiotic division, the centromeres of homologous chromosomes get separated and molecular markers located close to the centromere tend to be reduced. Specifically, this means if the mother is heterozygous at a marker close to the centromere, the two alleles will separate in meiosis I and the two sister chromatids within each daughter cell will be homozygous. Whenever an uneven number of cross-overs between the centromere and the molecular marker occur, the two alleles separate in the second meiotic division (Johnson et al. 1996).

Triploid individuals may carry two chromatids of a single meiosis (a so-called half-tetrad; Zhao and Speed 1998). The supernumerary haploid chromosome set originates either from the mother (digyny) or the father (diandry) and may arise from non-disjunction of homologous chromosomes at meiosis I or by non-disjunction of sister chromatids at meiosis II. Diandric triploidies can also result from dispermy, the fertilization of a single egg by two sperm cells (Jacobs and Morton 1977). Since dispermy is not caused by a meiotic failure, a half-tetrad cannot be recovered and, hence, those triploids are not suitable for centromere mapping.

**Fig. 1:**
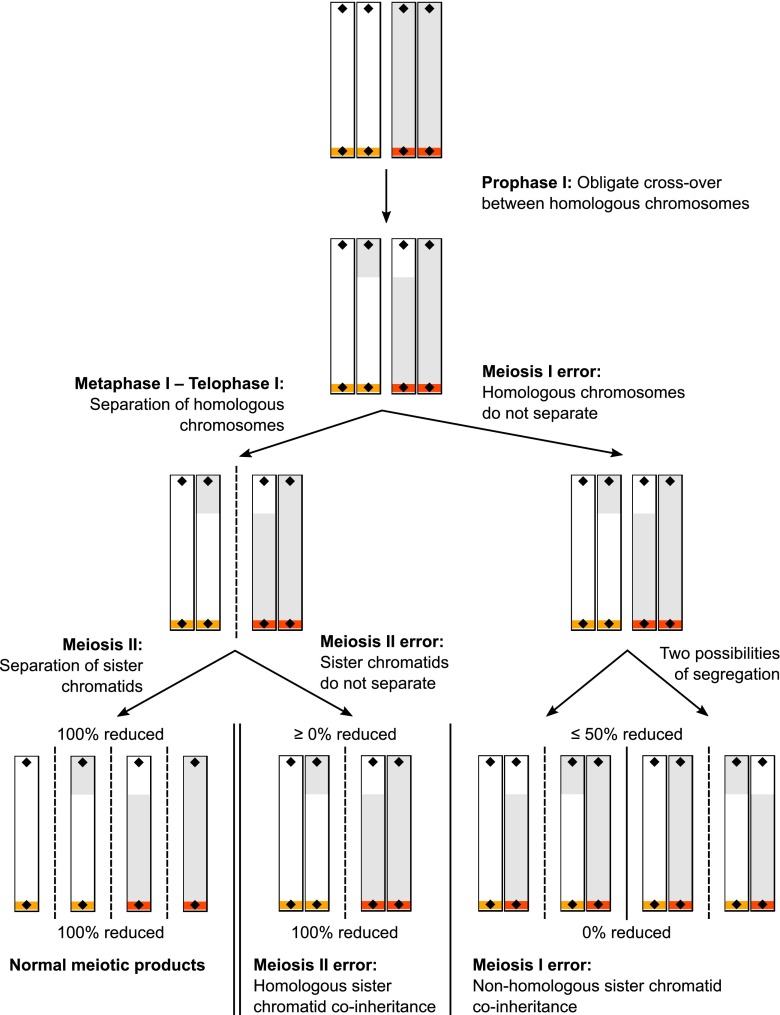
Schematic explanation of the centromere-marker-mapping approach in half-tetrads using triploids resulting from either a meiosis I or II error. The representation starts at early prophase I, at which stage the homologous chromosomes have been duplicated (each consisting of two sister chromatids) and synapsed. The two homologous chromosomes are depicted in *white* and *grey* and their centromeres in *orange* and *red*. In this scheme, the chromosome is acrocentric and thus a representation of all microchromosomes in the zebra finch genome. *Black diamonds* indicate the positions of two genetic markers (microsatellites) as they have been used in this study. During prophase I, at least one cross-over happens to ensure proper segregation of the homologous chromosomes. The normal meiotic process is depicted on the left hand side, resulting in four haploid (reduced) gametes (*bottom left*). Whenever a meiosis I error occurs, the homologous chromosomes do not separate. This results in two out of the four possible diploid gametes depicted on the *bottom right* (and two gametes containing no chromosomes). All four gametes are not reduced at the centromere, meaning that at a heterozygous microsatellite, both alleles will be passed on. A microsatellite at the distal telomeric end will be reduced in 50 % of the cases, if exactly one cross-over happens per chromosome and less if more than one cross-over occurs. Whenever a meiosis II error occurs, the sister chromatids do not separate. This results in one or both of the two diploid gametes depicted in the *bottom middle* (and either one gamete containing no chromosomes and two normal haploid gametes or two gametes containing no chromosomes, respectively). A heterozygous microsatellite completely linked to the centromere will always be reduced, meaning that only one of its two alleles will be passed on. A microsatellite at the distal telomeric end will never be reduced, if exactly one cross-over happens per chromosome and more often if more than one cross-over occurs. Thus, after identifying all inheritance events of a half-tetrad (maternal triploidies) and subsequently distinguishing between meiosis I and meiosis II errors using microsatellites at known centromeres, this knowledge can be transferred to chromosomes with unknown centromere positions to identify the chromosome end at which the centromere resides. The same logic applies to the sex chromosomes: In case of a meiosis I error, females will pass on one Z and one W chromosome; in case of a meiosis II error, females will either pass on two Z or two W chromosomes

Centromere-marker-mapping requires distinguishing between non-disjunction at the first or second meiotic division. In zebra finches, where some centromere positions are known, this can be done by genotyping a molecular marker close to these centromeres (Chakravarti and Slaugenhaupt 1987). Whenever the parent contributing the third chromosome set is heterozygous for the centromeric marker, the triploid offspring inherits both alleles (i.e. both homologous chromosomes) in case of a meiosis I error and two copies of the same allele (i.e. both sister chromatids) in case of an error in the second meiotic division. A molecular marker at the distal end may convey additional information, if exactly one or an uneven number of cross-overs happens between itself and the centromere, because then, an error in the second meiotic division would always lead to the inheritance of both alleles (because of the cross-over, the sister chromatids carry parts of both homologous chromosomes at that position; Côté and Edwards 1975). The logic of this centromere-marker-mapping strategy is depicted in Fig. 1.

In order to obtain several half-tetrads per chromosome, we use naturally occurring triploid zebra finches, which usually die at the embryo stage (Forstmeier and Ellegren 2010) but occasionally survive to adulthood (Girndt et al. 2014). By genotyping each triploid individual at one microsatellite close to the ten known centromeres and one close to a distal end on each of these chromosomes, we were able to distinguish digynic from diandric triploidies and subsequently identify whether the digynic meiotic failures occurred in the first or second meiotic division. Since all remaining chromosomes with an unknown physical centromere position are acrocentric (Pigozzi 2008). we then designed primers for microsatellites on both ends of each chromosome. Since one of the two markers was close to the centromere, we were able to orient the physical/linkage map in respect to the centromere for almost all assembled microchromosomes in the zebra finch genome.

## Material and methods

### Individuals and populations

When using microsatellites for identifying cases of triploidy, not all markers are expected to show three alleles because they (1) could be blind due to homozygosity of the parents or due to mother and father sharing the same alleles or (2) are located at a physical position along the chromosome that gets inherited twice from the same homolog (see “Introduction”). During the regular paternity analysis of 4993 alive birds (we consider them as alive birds if they hatch) and 2999 embryos (including cases where the egg shell broke or the egg was opened before the due date of hatching), 6 birds (3 of which survived to adulthood) and 28 embryos had been identified as being trisomic for at least three chromosomes (range 3–16), and we assumed that these 34 individuals were triploid. In previous studies using single nucleotide polymorphism (SNP) markers spread across the whole genome, a subset of these 34 individuals, namely 8 embryos (*n* = 1395 SNPs; Forstmeier and Ellegren 2010) and 2 adult birds (*n* = 2417 SNPs; Girndt et al. 2014). were confirmed as being triploid (trisomic for all 32 chromosomes in the WUSTL v3.2.4 assembly). An additional three embryos were found to be triploid by genotyping the same SNP set as in Girndt et al. (2014) in 115 embryos that had died from natural causes. Thus, in total, we had 37 individuals that were triploid. To determine whether the supernumerary haploid chromosome was inherited from the mother or the father, we included all the parents of the triploid individuals in our study.

The 37 triploid individuals stemmed from three different populations: (1) Our main population held at the Max Planck Institute for Ornithology in Seewiesen (*n* = 19; study population 18 in Forstmeier et al. 2007), (2) a recently wild-derived population held at the Max Planck Institute for Ornithology in Seewiesen (*n* = 13; originating from study population 4 in Forstmeier et al. 2007), (3) a population that was produced by crossing individuals from a captive population held in Cracow (study population 11 in Forstmeier et al. 2007) with our main population (*n* = 5). Since we used differing microsatellite sets for trisomy detection within and between each of the three populations, detection probabilities varied and a comparison of the rate of triploidy between populations is not meaningful. The only unbiased estimate of the rate of triploidy can be obtained from the 115 dead embryos genotyped with 2417 SNPs (Girndt et al. 2014), which yielded three triploids (2.6 %) among naturally dying embryos (with about 25–30 % of all embryos dying naturally during development).

### Genetic markers

For each of the ten chromosomes with a known centromere location, we designed primers to amplify two microsatellites, one of them located close to the known centromere and the other at the most distant chromosome end. On chromosome *Tgu5* and *Tgu6*, the FISH probes mapping closest to the centromere are located on sequences, whose positions within the chromosomes are not known (chromosomes *Tgu5_random* and *Tgu6_random*; Warren et al. 2010). Thus, we designed primers for microsatellites that are positioned on the same Contig as the FISH probes. Yet on chromosome *Tgu6_random*, the marker appears to be quite far from the centromere, so we designed an additional primer pair for a microsatellite on chromosome *Tgu6* which should be located close to the centromere. For the 22 microchromosomes with an unknown centromere location, we designed primers for two microsatellites, one at the start and one at the end of each chromosome (excluding the difficult-to-assemble chromosome *Tgu16* which is only 9.9 kb in the current genome assembly but known to be several hundred times larger; Ekblom et al. 2011; Pichugin et al. 2001). Since all chromosomes with an unknown centromere position are acrocentric (Pigozzi 2008). one microsatellite should be located close to the centromere and the other one close to the distal end (see Supplementary Table S1 for detailed information for each primer pair). However, if parts of the chromosome are missing from the assembly, markers could be further away from the centromere or from the distal end (see “Discussion”).

We used the primer pair 3007/3112 for sexing all embryos, which amplifies an intron in the *CHD1* gene differing in length on chromosome *TguZ* and chromosome *TguW* (Ellegren and Fridolfsson 1997).

### DNA extraction and genotyping

DNA was extracted from blood or tissue samples of all triploid individuals and their parents using the NucleoSpin Blood QuickPure Kit (Macherey-Nagel). Both the Type-it Microsatellite PCR Kit (Qiagen) and the Multiplex PCR Kit (Qiagen) were used for genotyping following manufacturer’s instructions (with the exception of an extension step of 60 °C for 30 min instead of 72 °C for 10 min with the Multiplex PCR Kit). Details on the PCR protocol for each multiplex are given in Supplementary Table S1.

### Determination of parental origin

We first determined whether the supernumerary haploid chromosome set was inherited from the mother or the father. For that, we considered those markers as being informative which showed the genotype AB in one parent, CD or CC in the other parent and ABC or ABD in the offspring. The parental origin of the additional chromosome set could be determined in 32 out of the 37 individuals with at least two markers per individual being informative (Supplementary Table S2). Of the remaining five individuals, two were found to be tetraploid with one additional chromosome set inherited from the mother and one from the father, and were hence still useful for the current study (2011_180, 2011_251). The other three individuals (K2012/13_125, 2011_289, 2006_584) had to be excluded because they were uninformative at all marker loci or appeared to be a mixture of digynic and diandric origins of the third chromosome set.

### Determination of mechanism of origin

Triploidy may arise from the non-disjunction of homologous chromosomes at meiosis I or by non-disjunction of sister chromatids at meiosis II. Since diandric triploidies may also result from dispermy, in which case a half-tetrad cannot be recovered, they are not useful for centromere mapping and were excluded from further analyses and will be described elsewhere (*n* = 12).

**Fig. 2:**
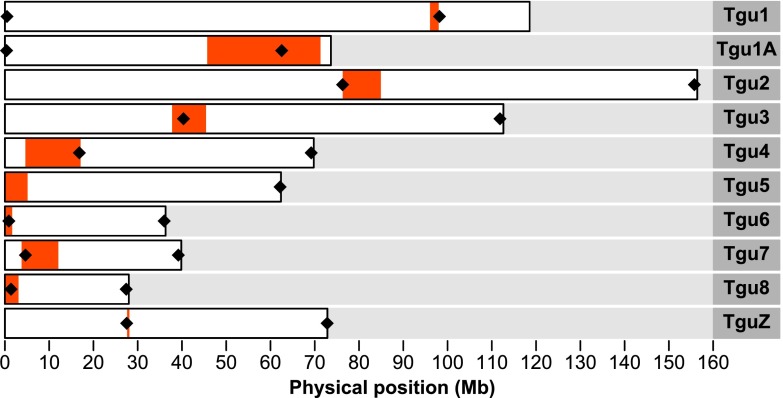
The ten chromosomes in the current zebra finch genome assembly (WUSTL v3.2.4) with a known centromere position in reference to the physical map. The centromere positions have been inferred by FISH (Warren et al. 2010). The intervals between the FISH probes closest flanking the centromeres are indicated in *red. Black diamonds* indicate the positions of microsatellite markers used in this study. On chromosome *Tgu5*, the microsatellite marker proximal to the centromere is located on *Tgu5_random* and thus not indicated in the figure (see main text for an explanation). Chromosome nomenclature follows the one introduced by Itoh and Arnold (2005) and Warren et al. (2010)

In the remaining 20 digynic triploids and the two tetraploids (2011_180, 2011_251), those markers located close to the known centromeres on the ten largest chromosomes (*Tgu1*–*Tgu8*, *Tgu1A* and *TguZ*) were used to distinguish between the non-disjunction of homologous chromosomes at meiosis I or non-disjunction of sister chromatids at meiosis II (Fig. 2). For that purpose, we assumed that the centromeric markers were in complete linkage with the centromere. Hence, whenever the mother was heterozygous at a centromeric marker and passed on both her alleles to the triploid offspring, we took it as evidence for an error in the first meiotic division. Each time she passed on only one of her two alleles, it was pointing to an error in meiosis II (see “Introduction” and Fig. 1 for the underlying logic).

Female birds carry one Z and one W chromosome. In zebra finches, the Z and the W chromosome pair during meiosis I (Pigozzi and Solari 1998) and a mandatory recombination event happens in the pseudoautosomal region (PAR) (Pigozzi 2008). Since the PAR is located at one end of chromosome *TguZ* (minimum range 1,213,256–1,464,488 bp; Stapley et al. 2008) and the centromere is located around 28 Mb, a centromeric marker will always be located on chromosome *TguZ* and not recombine with chromosome *TguW*. If non-disjunction happens in meiosis I, females will always inherit a single Z and a single W chromosome. Meiosis II errors should lead to the inheritance of either two Z or two W chromatids with equal probabilities.

### Mapping of centromeres

We used the maximum likelihood method in Chakravarti et al. (1989) to estimate the genetic distance of our markers to the centromere under complete interference, i.e. that only a single cross-over between the marker and the centromere is allowed. Complete interference is a reasonable assumption since usually a single cross-over happens per chromosome arm in the zebra finch (Calderón and Pigozzi 2006). However, one should keep in mind that the estimated genetic distances are restricted to 50 cM (if there is one cross-over in any meiosis between two markers then they are 50 cM apart) and may be underestimated because of occasional double or triple cross-overs.

In order to estimate the genetic distance of our markers from the centromere, we define *m*_1_ as being the number of non-reduced triploid individuals and *m*_2_ being the number of reduced triploid individuals at a specific marker resulting from an error in meiosis I and *m* = *m*_1_ + *m*_2_. Similarly, we define *n*_1_ as being the number of non-reduced triploid individuals and *n*_2_ being the number of reduced triploid individuals at a specific marker resulting from an error in meiosis II and *n* = *n*_1_ + *n*_2_. Then, we calculated the maximum likelihood estimate of *y*, the probability of a recombinant meiotic tetrad, by solving the equation (*m* + *n*) × *y*^2^ − (3 × (*m* + *n*) − (2*m*_1_ + *n*_2_)) × *y* + 2 × (*m*_2_ + *n*_1_) = 0. The variance in *y* is given by Var(*y*) = *y* × (1 − *y*) × (2 − *y*)/(*n* + (*m* + *n*) × (1 − *y*)) (Chakravarti et al. 1989). By assuming complete cross-over interference, *y* can be translated into the marker-centromere distance (*w*; in cM) with *w* = *y*/2 × 100 (Chakravarti and Slaugenhaupt 1987). The variance in *w* is given by Var(*w*) = Var(*y*)/4 × 100 (Deka et al. 1990).

The locations of several microsatellite markers were not covered by the published linkage map (Backström et al. 2010). Thus, we inferred the genetic location of those microsatellites by extrapolating linearly from the closest two markers in the linkage map.

## Results

### Parent and mechanism of origin

Twenty out of the 37 triploid individuals inherited the supernumerary haploid chromosome set from their mother with at least three markers per individual indicating an error in the maternal meiosis (and no marker indicating an error in the paternal meiosis; Supplementary Table S2). Two additional individuals were tetraploid, with one additional chromosome set passed on from their mother and one from their father (dispermy). For the purpose of this study, we will refer to these two individuals subsequently as digynic triploid since the additional paternal chromosome set is not of relevance for centromere mapping.

In 12 out of the 22 digynic triploid cases, the error occurred in the first meiotic division and in 10 cases it occurred in the second meiotic division (Table 1). One of those ten triploid individuals (individual G8-3-4) could not be assigned with maximal confidence to be the result of an error in meiosis II. Since seven out of eight informative chromosomes indicated an error in the second meiotic division, a cross-over on chromosome *Tgu8* between the centromeric marker (located 1.38 Mb from the chromosome end) and the centromere seems the most parsimonious explanation (Table 1). As expected, all triploid individuals resulting from an error in female meiosis I inherited both a Z and a W chromosome, and those triploids originating from an error in the second meiotic division either got two Z or presumably two W chromosomes (we have no markers on chromosome *TguW* to prove the presence of two W chromosomes). For two individuals, the status of the sex chromosomes could not be inferred unambiguously but was consistent with the expectation (individuals B2013_088 and G8-3-4; Table 1).

**Table 1:** Digynic triploid individuals resulting from non-disjunction in the first or second meiotic division with their sex chromosome karyotype and the numbers of reduced and non-reduced markers at known centromeres and distal ends (from nine chromosomes as in Fig. 2 except chromosome *TguZ*).

Individual ID	Sex chromosome^a^	Known centromere			Known distal end			Meiotic error
		Not reduced	Reduced	Not informative	Not reduced	Reduced	Not informative	
B2012_130	*ZZW*	6	**0**	3	1	2	6	MI
B2011_258a	*ZZW*	5	**0**	4	4	4	1	MI
B2013_088	*ZZW*/*ZWW*	5	**0**	4	4	0	5	MI
B2013_207	*ZZW*	5	**0**	4	4	0	5	MI
B2012_129	*ZZW*	5	**0**	4	1	2	6	MI
B2013_086	*ZZW*	4	**0**	5	2	4	3	MI
B2013_198	*ZZW*	4	**0**	5	3	2	4	MI
2006_486	*ZZW*	4	**0**	5	3	1	5	MI
2011_328	*ZZW*	4	**0**	5	3	1	5	MI
2011_183	*ZZW*	4	**0**	5	1	2	6	MI
B2011_017	*ZZW*	1	**0**	8	4	1	4	MI
2011_180	*ZZZW*	3	**0**	6	5	2	2	MI + polyspermy
2006_550	*ZZZ*	**0**	7	2	5	1	3	MII
B2013_236	*ZWW*	**0**	7	2	3	2	4	MII
B2011_187	*ZWW*	**0**	6	3	5	3	1	MII
B2013_227	*ZWW*	**0**	6	3	2	4	3	MII
G12-1-1	*ZWW*	**0**	6	3	4	2	3	MII
2011_205	*ZZZ*	**0**	5	4	4	2	3	MII
2005_118	*ZWW*	**0**	5	4	4	1	4	MII
2011_308	*ZZZ*	**0**	4	5	3	2	4	MII
G8-3-4	*ZWW*/*ZZW*	**1** ^b^	7	1	6	3	0	MII
2011_251	*ZZZZ*	**0**	5	4	2	1	6	MII + polyspermy

Bold print highlights the key observation for inferring errors in the first meiotic division (MI) versus the second meiotic division (MII). These individuals and the information about MI or MII errors were subsequently used for mapping the location of centromeres on additional chromosomes

^a^In contrast to the Z chromosome, we only have markers that determine the presence of a W chromosome but not any polymorphic markers to distinguish the presence of one versus two W chromosomes, so the latter was inferred by logic whenever possible

^b^Since seven chromosomes indicate an error in meiosis II, a cross-over between the marker and the centromere on chromosome *Tgu8* is the most parsimonious explanation

### Centromere positions and comparison to the linkage map

**Fig. 3:**
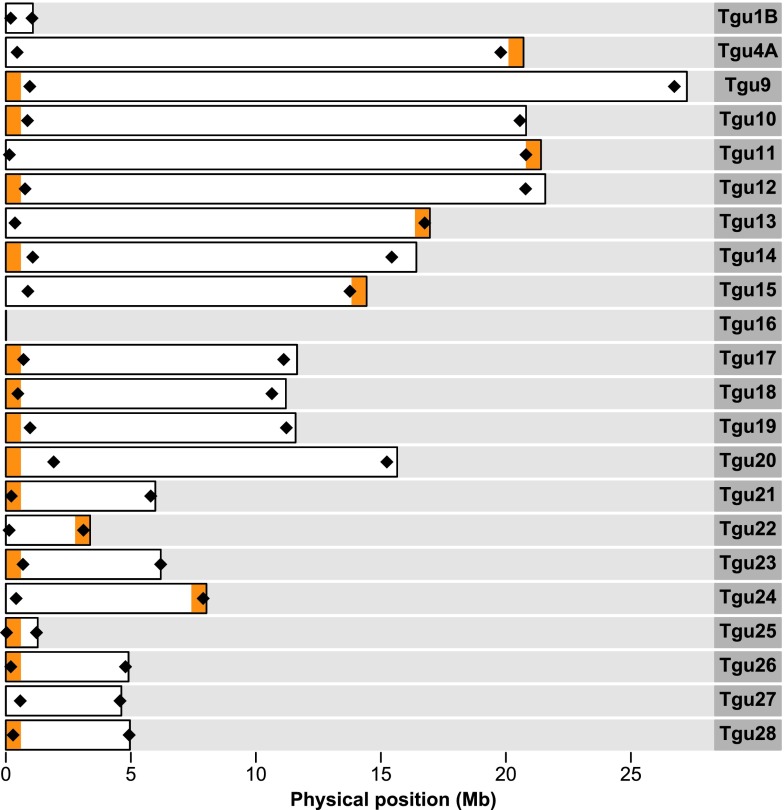
The 22 acrocentric chromosomes in the current zebra finch genome assembly (WUSTL v3.2.4) with an unknown centromere position in reference to the physical map. For 19 of these chromosomes, the positions of the centromeres were mapped and are indicated in *orange*. For clarity, each centromere position is indicated by a 600-kb wide interval, which does not reflect the true extent of the centromere though. *Black diamonds* indicate the positions of microsatellite markers used in this study. Chromosome nomenclature follows the one introduced by Itoh and Arnold (2005) and Warren et al. (2010)

Female non-disjunction in both the first and second meiotic divisions is informative for centromere mapping. For each chromosome, we had 2–16 informative meioses at the centromere and in total 8–39 informative inheritance events at the centromere and the distal end taken together. We hence were able to determine the location of centromeres on all but three chromosomes (these three were chromosomes *Tgu1B* and *Tgu16* and *Tgu27*; Fig. 3 and Table 2). The centromeric markers on chromosomes *Tgu8*, *Tgu13*, *Tgu21* and *Tgu25* were not completely linked to the centromere (especially on chromosome *Tgu13*), yet the distal end markers contained enough information to localize the centromere unambiguously (the two markers on these chromosomes were at least 18.34 cM apart).

**Table 2:** Physical and genetic position of all microsatellite markers used in this study. The genetic position and its standard deviation are in reference to the centromere and were calculated from the numbers of reduced and not reduced chromosomes in meiosis I and II (Chakravarti et al. 1989)

Chromosome	Marker	Position (Mb)	MI error			MII error			Position (cM ± SD)	Linked cytogenetic feature
			Not reduced	Reduced	Not informative	Not reduced	Reduced	Not informative		
*Tgu1*	1_cen_98.17	98.17	4	**0**	8	**0**	4	6	0.00	Centromere
*Tgu1A*	1A_cen_62.53	62.53	7	**0**	5	**0**	4	6	0.00	Centromere
*Tgu2*	2_cen_76.29	76.29	1	**0**	11	**0**	7	3	0.00	Centromere
*Tgu3*	3_cen_40.34	40.34	7	**0**	5	**0**	9	1	0.00	Centromere
*Tgu4*	4_cen_16.82	16.82	6	**0**	6	**0**	8	2	0.00	Centromere
*Tgu4A*	4A_en_19.79	19.79	1	**0**	11	**0**	3	7	0.00	Centromere
*Tgu5_random*	5rand_cen_0.26	0.26	7	**0**	5	**0**	8	2	0.00	Centromere
*Tgu6*	6_cen_0.89	0.89	6	**0**	6	**0**	5	5	0.00	Centromere
*Tgu7*	7_cen_4.65	4.65	2	**0**	10	**0**	8	2	0.00	Centromere
*Tgu8*	8_cen_1.38	1.38	10	**0**	2	**1**	5	4	4.65 ± 4.43	Centromere
*Tgu9*	9_st_0.96	0.96	2	**0**	10	**0**	0	10	0.00	Centromere
*Tgu10*	10_st_0.86	0.86	10	**0**	2	**0**	6	4	0.00	Centromere
*Tgu11*	11_en_20.8	20.8	4	**0**	8	**0**	7	3	0.00	Centromere
*Tgu12*	12_st_0.77	0.77	4	**0**	8	**0**	5	5	0.00	Centromere
*Tgu13*	13_en_16.75	16.75	3	**5**	4	**2**	5	3	27.04 ± 8.08	Centromere
*Tgu14*	14_st_1.07	1.07	6	**0**	6	**0**	8	2	0.00	Centromere
*Tgu15*	15_en_13.76	13.76	1	**0**	11	**0**	3	7	0.00	Centromere
*Tgu17*	17_st_0.7	0.7	8	**0**	4	**0**	7	3	0.00	Centromere
*Tgu18*	18_st_0.48	0.48	1	**0**	11	**0**	6	4	0.00	Centromere
*Tgu19*	19_st_0.97	0.97	5	**0**	7	**0**	3	7	0.00	Centromere
*Tgu20*	20_st_1.91	1.91	2	**0**	10	**0**	5	5	0.00	Centromere
*Tgu21*	21_st_0.22	0.22	6	**1**	5	**1**	7	2	8.53 ± 5.63	Centromere
*Tgu22*	22_en_3.1	3.1	5	**0**	7	**0**	1	9	0.00	Centromere
*Tgu23*	23_st_0.68	0.68	9	**0**	3	**0**	6	4	0.00	Centromere
*Tgu24*	24_en_7.89	7.89	5	**0**	7	**0**	4	6	0.00	Centromere
*Tgu25*	25_st_0.03	0.03	3	**1**	8	**0**	7	3	5.31 ± 5.17	Centromere
*Tgu26*	26_st_0.2	0.2	3	**0**	9	**0**	5	5	0.00	Centromere
*Tgu28*	28_st_0.29	0.29	9	**0**	3	**0**	6	4	0.00	Centromere
*TguZ*	Z_cen_27.51	27.51	3	**0**	9	**0**	4	6	0.00	Centromere
*Tgu1*	1_st_0.48	0.48	5	2	5	2	5	3	18.28 ± 7.72	Distal end
*Tgu1A*	1A_st_0.38	0.38	8	2	2	5	1	4	35.84 ± 7.86	Distal end
*Tgu2*	2_en_155.77	155.77	6	0	6	4	5	1	17.61 ± 7.09	Distal end
*Tgu3*	3_en_111.84	111.84	2	1	9	3	3	4	26.16 ± 9.46	Distal end
*Tgu4*	4_en_69.2	69.2	3	2	7	3	5	2	22.60 ± 7.96	Distal end
*Tgu4A*	4A_st_0.45	0.45	4	5	3	3	0	7	50.00	Distal end
*Tgu5*	5_en_62.17	62.17	5	5	2	7	0	3	50.00	Distal end
*Tgu6*	6_en_35.99	35.99	3	2	7	7	0	3	50.00	Distal end
*Tgu7*	7_en_39.18	39.18	2	6	4	6	1	3	45.58 ± 5.13	Distal end
*Tgu8*	8_en_27.41	27.41	1	1	10	1	2	7	22.98 ± 12.95	Distal end
*Tgu9*	9_en_26.74	26.74	2	0	10	5	1	4	39.37 ± 8.12	Distal end
*Tgu10*	10_en_20.56	20.56	2	5	5	6	0	4	50.00	Distal end
*Tgu11*	11_st_0.14	0.14	3	4	5	5	3	2	35.51 ± 7.33	Distal end
*Tgu12*	12_en_20.79^a^	20.79	0	1	11	0	0	10	50.00	Distal end
*Tgu13*	13_st_0.37	0.37	2	6	4	7	0	3	50.00	Distal end
*Tgu14*	14_en_15.44	15.44	5	6	1	6	0	4	50.00	Distal end
*Tgu15*	15_st_0.88	0.88	5	4	3	6	0	4	50.00	Distal end
*Tgu17*	17_en_11.11	11.11	0	3	9	2	1	7	41.67 ± 10.06	Distal end
*Tgu18*	18_en_10.64	10.64	1	4	7	4	0	6	50.00	Distal end
*Tgu19*	19_en_11.22	11.22	3	5	4	3	0	7	50.00	Distal end
*Tgu20*	20_en_15.24	15.24	1	3	8	5	0	5	50.00	Distal end
*Tgu21*	21_en_5.8	5.8	0	2	10	2	0	8	50.00	Distal end
*Tgu22*	22_st_0.13	0.13	4	2	6	8	0	2	50.00	Distal end
*Tgu23*	23_en_6.19	6.19	2	4	6	1	5	4	23.38 ± 8.77	Distal end
*Tgu24*	24_st_0.41	0.41	4	5	3	7	0	3	50.00	Distal end
*Tgu25*	25_en_1.22	1.22	4	0	8	5	4	1	24.12 ± 7.76	Distal end
*Tgu26*	26_en_4.78	4.78	1	6	5	5	1	4	45.52 ± 5.57	Distal end
*Tgu28*	28_en_4.93	4.93								Distal end
*TguZ*	Z_en_72.81	72.81	10	0	2	0	9	1	0.00	Distal end
*Tgu1B*	1B_en_1.05	1.05	5	4	3	2	5	3	23.57 ± 7.85	
*Tgu1B*	1B_st_0.19	0.19	1	7	4	0	6	4	26.27 ± 8.53	
*Tgu27*	27_st_0.58	0.58	1	1	10	0	0	10	50.00	
*Tgu27*	27_en_4.57	4.57	0	5	7	0	0	10	50.00	

A genetic position of 0 cM indicates complete linkage to the centromere. Bold print highlights the key observation for inferring linkage to the centromere

^a^The microsatellite 12_en_20.79 is duplicated in the genome. Since we do not know whether the second copy is also located on chromosome *Tgu12*, it is not possible to infer triploidy by the occurrence of all alleles from a parent. Yet the marker is informative if only a single allele gets inherited (because then it was reduced)

**Fig. 4:**
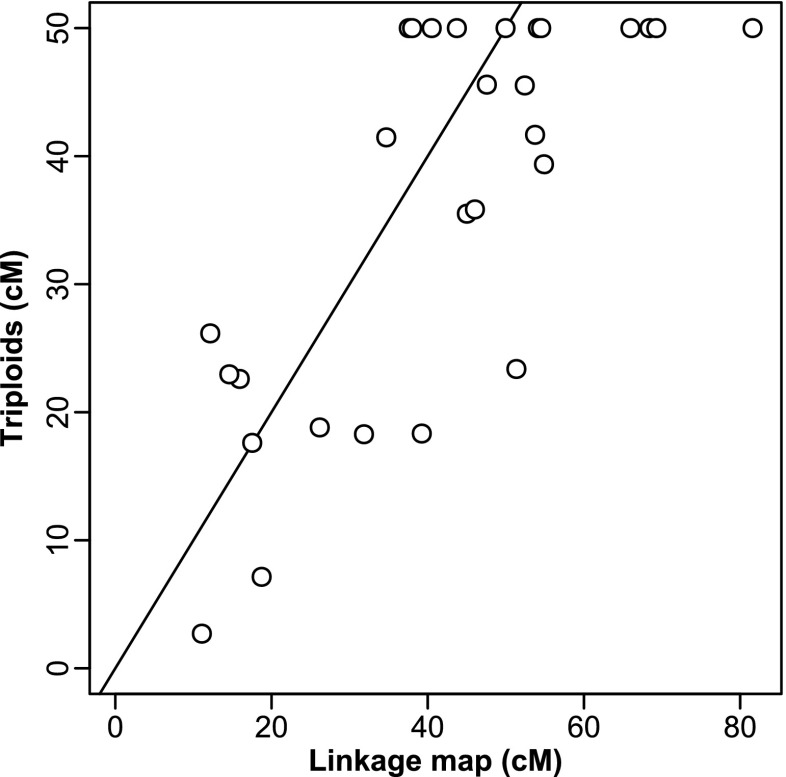
Comparison of genetic map distances between the two microsatellite markers for each chromosome taking estimates from the linkage map (Backström et al. 2010) and estimates from the here presented marker-centromere-mapping using triploids. The *line* represents the diagonal line of equality

We compared the genetic distance between the two markers on each chromosome estimated from the published linkage map with the here estimated genetic distance from the centromere-marker-mapping. The correlation was highly significant (Pearson’s *r* = 0.75, 95 % confidence interval 0.53–0.88, *df* = 26, *P* = 4 × 10^−6^; Fig. 4), even though the estimated genetic distances between markers from the centromere-marker-mapping are restricted to be maximally 50 cM (see “Material and methods”).

## Discussion

We here make use of naturally occurring triploid zebra finches to map the location of centromeres in reference to the physical genome assembly. By using centromere-marker-mapping techniques, we were able to map the centromere position on almost all of the 32 assembled chromosomes in the current zebra finch genome assembly (WUSTL v3.2.4).

### Problematic cases and discrepancies from expectations

Völker et al. (2010) used FISH mapping on chromosome *Tgu4A* and localized the centromere on the opposite side of the chromosome than we did. If the centromere was indeed located on the side Völker et al. (2010) suggested, it would introduce 11 errors in our centromere mapping (5 in meiosis I and 6 in meiosis II triploids) and the genetic positions of microsatellites should switch from 0 to 50 cM, making a mapping error extremely unlikely. Thus, we suspect that either populations differ in respect to their centromere position on chromosome *Tgu4A* or that Völker et al. (2010) identified the wrong chromosomal side, which may happen on acrocentric chromosomes if centromeres are not specifically stained.

For chromosome *Tgu9*, we had only two informative meioses at the centromere, yet these two meioses indicated perfect linkage of our marker with the centromere and the second marker on chromosome *Tgu9* is located 39 cM away from the centromere. Chromosome *Tgu9* is acrocentric (Pigozzi 2008) and its linkage map spans almost the whole assembled chromosome (Backström et al. 2010). Thus, the genetic positions from the centromere-marker-mapping should correspond to the linkage map positions in Backström et al. (2010), and they agree reasonably well (0 vs 0.3 cM and 39 vs 55 cM for the first and second microsatellite markers, respectively). Thus, also for chromosome *Tgu9*, we are confident that we localized the centromere at the correct end of the chromosome. All other chromosomes had at least four informative meioses at the centromere, indicating high reliability of our mapping results.

From estimates of the repeat content of the zebra finch genome, it seems possible that the main satellite sequences are still missing from the genome assembly (Warren et al. 2010). In line with this, the smallest microchromosomes in chicken are at least 3.4 Mb in size as measured by pulse-field electrophoresis (Pichugin et al. 2001), and this is probably also true for the zebra finch given that the karyotype and genome size between the two species are highly conserved (Peterson et al. 1994; Pigozzi and Solari 1998). There are three chromosomes which are shorter than 3.4 Mb in the zebra finch reference genome, namely *Tgu1B*, *Tgu16* and *Tgu25* (1.08 Mb, 9 kb and 1.28 Mb, respectively). For chromosome *Tgu16*, we did not even attempt to map the centromere since more than 99 % of this difficult-to-assemble chromosome is missing from the assembly. Chromosome *Tgu1B*, in our linkage map, appears to be linked to chromosome *Tgu1* (Backström et al. 2010) and it thus should not even have a centromere. Our failure to map its centromere might be regarded as further support that chromosome *Tgu1B* is not an independent chromosome. On chromosome *Tgu25*, our centromeric marker was estimated to be 5.3 cM away from the actual centromere. Given that some parts of the chromosome are absent from the reference assembly (the terminal marker in our linkage map at the centromeric side of the chromosome maps to *Tgu25_random*; Backström et al. 2010), even markers at the end of each chromosome could be separated from the centromere and cross-overs may occasionally happen between the marker and centromere. This is probably also the case for chromosomes *Tgu8*, *Tgu13* and *Tgu21*. For chromosomes *Tgu8* and particularly *Tgu13*, we have direct evidence from our linkage map that at the centromeric side of the chromosome, parts are missing in the reference assembly because the terminal markers are located on *Tgu8_random* and *Tgu13_random*, respectively (Backström et al. 2010). Chromosome *Tgu21* is among those chromosomes with the most amount of sequence unordered on its random chromosome, indicating that also sequence between the centromere and the marker may be missing.

Similarly, even though the microsatellite markers on the acrocentric chromosomes were located at most only 1.91 Mb from the chromosome ends, several chromosomes are estimated to be genetically shorter than 50 cM, which is the minimum genetic size of a chromosome, since at least one cross-over is required for proper chromosome segregation (Petronczki et al. 2003). First, this could be due to the fact that there is subtelomeric sequence missing in the current genome assembly (Warren et al. 2010). Second, with only two markers per chromosome, we were unable to identify double cross-overs and could not distinguish single cross-overs from triple cross-overs which leads to an underestimation of the genetic length of a chromosome (Danzmann and Gharbi 2001).

### Origin of triploidies

Triploidy is one of the most common chromosome abnormalities in spontaneous human abortions, estimated to occur in 1–2 % of all conceptions (Jacobs et al. 1982). In their study on zebra finches, Forstmeier and Ellegren (2010) found 4 triploids among 331 embryos that died during development (1.2 %), and in an independent sample from the same population of 115 embryos that died during development, we found 3 triploids (2.6 %), which is not significantly different from the first estimate (Fisher’s exact test *P* = 0.38) and similar to rates found in chicken (1.6 vs 2.7 % in zebra finch and chicken (Thorne et al. 1991). respectively, Fisher’s exact test *P* = 0.25).

In humans, the relative importance of diandric to digynic triploidies is still a matter of debate, probably resulting from ascertainment bias and differing sampling schemes (Zaragoza et al. 2000). Estimates for diandric origin range from around 20 to 89 % of all triploidies with a mean of 64.4 % (Joergensen et al. 2014; McFadden et al. 1993; McFadden and Langlois 2000; McFadden and Robinson 2006 and references therein). Digynic triploidies result from errors both in the first and second meiotic divisions with a slight bias towards errors in the second meiotic division (51 vs 63 cases, respectively; calculated from Joergensen et al. 2014; McFadden et al. 1993; McFadden and Langlois 2000; McFadden and Robinson 2006 and references therein). In our sample of 34 triploid zebra finches, 41.2 % had a diandric origin and the 22 digynic triploidies resulted from errors in meiosis I and II with about equal frequencies. Thus, digynic meiotic errors may be a more common cause of triploidy in zebra finches than in humans (Fisher’s exact test *P* = 0.004), which is also the case in chicken (Fechheimer 1981). Rates of meiosis I and II errors were similar between humans and zebra finches, contrasting results in chicken where errors in meiosis II seem to predominate (Bloom 1972; Fechheimer 1981; Thorne et al. 1991).

### Utility for future studies

We here report the approximate location of an additional 19 centromeres in the zebra finch reference genome, meaning that in total, 29 of the 32 assembled chromosomes can now be oriented according to their centromere position. In genome scans for relative divergence between populations or species, centromeres often stand out as regions of increased differentiation (for example Ellegren et al. 2012), which has been interpreted either as signs for adaptive divergence (‘islands of speciation’), meiotic drive or background selection in the absence of recombination (reviewed in Cruickshank and Hahn 2014). All three processes lead to a reduction in diversity near centromeres, leaving the sign of a selective sweep. Bird genomics recently gained popularity (for example Jarvis et al. 2014; Zhang et al. 2014), and the zebra finch genome is commonly used as a reference assembly for other bird species. Thus, the newly developed centromeric and distal telomeric microsatellite markers can now be used to understand the selective forces shaping the genomic landscapes of diversity and divergence in more detail, for example in studies of meiotic drive or species divergence (Axelsson et al. 2010; Ellegren et al. 2012; Knief et al. 2015).

## Electronic supplementary material

Below is the link to the electronic supplementary material.

ESM 1 Supplementary Table S1 (PDF 240 kb)

ESM 2 Supplementary Table S2 (PDF 30 kb)
