# Supplementary material for: Mapping centromeres of microchromosomes in the zebra finch (Taeniopygia guttata) using half-tetrad analysis
Source: Chromosoma. 2015 Dec 15;125(4):757–68. doi: 10.1007/s00412-015-0560-7 (PMC5023761; doi:10.1007/s00412-015-0560-7)
Supplement: Supplementary file 1 — Supplementary Table S1 (PDF 240 kb) [file 412_2015_560_MOESM1_ESM.pdf]

Supplementary Table S1: Description of primer pairs and PCR conditions for each microsatellite used in this study. The column 'Position (bp)' gives the location of microsatellites in the WUSTL v3.2.4 zebra finch genome assembly. For chromosomes with a previously known centromere position we indicated this in the column 'Location' and gave its position in the WUSTL v3.2.4 genome assembly in the column 'Centromere (Mb)'.

Chromosome	Location	Position (bp)	Primer Name	Tm pair (°C)	Primer sequence	Centromere (Mb)	Chromosome length (Mb)	Link to UCSC genome browser	Fluorescence label	Genotyping Kit	Multiplex	Volume for multiplex (µl)	Annealing temperature	Number of cycles	Chromosome length	Distance from end
Tgu1	start	477,304	1_st_0.48-F	59.35	TCAGGTTGAGATGGGCCTGT	96-98	118.55	chr1:477287-477321	NED	Type-it Microsatellite PCR	32	1	58	24	118548696	477,304
	start	477,304	1_st_0.48-R	59.35	GGGATTCTGGCAACACTGGA	96-98	118.55			Type-it Microsatellite PCR	32	1	58	24	118548696	477,304
Tgu1	centromere	98,173,031	1_cen_98.17-F	59.585	TGCTTGAACCACTTGGGACAC	96-98	118.55	chr1:98173002-98173060	6FAM	Type-it Microsatellite PCR	25	0.5	59	27	118548696	
	centromere	98,173,031	1_cen_98.17-R	59.585	AAGGCACAGTCTGGCAGGAA	96-98	118.55			Type-it Microsatellite PCR	25	0.5	59	27	118548696	
Tgu1A	start	375,707	1A_st_0.38-F	59.35	GGAGGCATCTTCGTGGGTTT	46-71	73.66	chr1A:375668-375745	PET	Type-it Microsatellite PCR	29	0.5	59	28	73657157	375,707
	start	375,707	1A_st_0.38-R	59.35	CGTGGCTTCTGCCTTCCATA	46-71	73.66			Type-it Microsatellite PCR	29	0.5	59	28	73657157	375,707
Tgu1A	centromere	62,529,285	1A_cen_62.53-F	60.375	AGGGAGAAAGAGGCCAGAGG	46-71	73.66	chr1A:62529265-62529304	VIC	Type-it Microsatellite PCR	25	0.5	59	27	73657157	
	centromere	62,529,285	1A_cen_62.53-R	60.375	GGGGAAGATGCACAGTTCCA	46-71	73.66			Type-it Microsatellite PCR	25	0.5	59	27	73657157	
Tgu1B	start	192,813	1B_st_0.19-F	59.35	GGAGCAGATTCCCATTGCTG		1.08	chr1B:192781-192845	VIC	Type-it Microsatellite PCR	4	4	59	26	1083483	192,813
	start	192,813	1B_st_0.19-R	59.35	TGGTGAGGTGCCCAAAACTC		1.08			Type-it Microsatellite PCR	4	4	59	26	1083483	192,813
Tgu1B	end	1,045,557	1B_en_1.05-F	57.87	GTTCATGCATCCTGCAAGTCA		1.08	chr1B:1045532-1045581	NED	Multiplex PCR	1	1	57	29	1083483	37,927
	end	1,045,557	1B_en_1.05-R	57.87	AGCTTGTTCCTTGGATCCTGA		1.08			Multiplex PCR	1	1	57	29	1083483	37,927
Tgu2	centromere	76,293,350	2_cen_76.29-F	57.585	GATGGGGAAAAGCAGCCAAT	76-85	156.41	chr2:76293320-76293380	PET	Type-it Microsatellite PCR	21	1	58	29	156412533	
	centromere	76,293,350	2_cen_76.29-R	57.585	CCCAGGCCAACCAATGTTAAA	76-85	156.41			Type-it Microsatellite PCR	21	1	58	29	156412533	
Tgu2	end	155,765,428	2_en_155.77-F	59.35	AGGGGAGTCCAAAGGCTGAA	76-85	156.41	chr2:155765380-155765476	NED	Type-it Microsatellite PCR	34	0.5	58	29	156412533	647,105
	end	155,765,428	2_en_155.77-R	59.35	TTTGGGAGGTAGCCCTTGCT	76-85	156.41			Type-it Microsatellite PCR	34	0.5	58	29	156412533	647,105
Tgu3	centromere	40,344,621	3_cen_40.34-F	59.585	CAGTCCTTTGCCTTGGATCAC	38-45	112.62	chr3:40344594-40344648	PET	Type-it Microsatellite PCR	24	0.5	59	27	112617285	
	centromere	40,344,621	3_cen_40.34-R	59.585	AATGGCTCAGGCTTGCTGTG	38-45	112.62			Type-it Microsatellite PCR	24	0.5	59	27	112617285	
Tgu3	end	111,842,386	3_en_111.84-F	59.35	GAGCACCACGTTCCCAACTT	38-45	112.62	chr3:111842333-111842439	PET	Type-it Microsatellite PCR	34	0.5	58	29	112617285	774,899
	end	111,842,386	3_en_111.84-R	59.35	CCAGGTGTTTTGGGCTGAGA	38-45	112.62			Type-it Microsatellite PCR	34	0.5	58	29	112617285	774,899
Tgu4	centromere	16,818,395	4_cen_16.82-F	59.82	TGCTTAATCCCACTGCTCTGC	5-17	69.78	chr4:16818378-16818411	NED	Type-it Microsatellite PCR	22	0.5	59	27	69780378	
	centromere	16,818,395	4_cen_16.82-R	59.82	GGAGCCCTAAATGCAATGGAG	5-17	69.78			Type-it Microsatellite PCR	22	0.5	59	27	69780378	
Tgu4	end	69,199,692	4_en_69.2-F	61.59	CAACAGCCCAGGAGAGCTGA	5-17	69.78	chr4:69199661-69199722	6FAM	Type-it Microsatellite PCR	30	0.5	60	28	69780378	580,687
	end	69,199,692	4_en_69.2-R	61.59	TCCCAGAGAGGGACTGACACA	5-17	69.78			Type-it Microsatellite PCR	30	0.5	60	28	69780378	580,687
Tgu4A	start	450,713	4A_st_0.45-F	59.35	AGCTTCAAACTGCGCTCTGC		20.70	chr4A:450690-450736	6FAM	Type-it Microsatellite PCR	33	1.5	60	27	20704505	450,713
	start	450,713	4A_st_0.45-R	59.35	CATGGACACCATAGCCAGCA		20.70			Type-it Microsatellite PCR	33	1.5	60	27	20704505	450,713
Tgu4A	end	19,794,737	4A_en_19.79-F	61.4	AGGGCAGGAAGTGGGTCTGT		20.70	chr4A:19794724-19794749	6FAM	Type-it Microsatellite PCR	21	0.5	58	29	20704505	909,769
	end	19,794,737	4A_en_19.79-R	61.4	GTTGTCTTCCCCTCCCCACT		20.70			Type-it Microsatellite PCR	21	0.5	58	29	20704505	909,769
Tgu5_random	centromere	260,603	5rand_cen_0.26-F	59.35	AATGTTCGGGTGAGGCACTG	0-5	62.37	chr5_random:260582-260624	NED	Type-it Microsatellite PCR	24	0.5	59	27	62374962	
	centromere	260,603	5rand_cen_0.26-R	59.35	GGGGAGCCCAATAACCTGAA	0-5	62.37			Type-it Microsatellite PCR	24	0.5	59	27	62374962	
Tgu5	end	62,172,843	5_en_62.17-F	59.35	TTGTCACCATCAGGGAGTGC	0-5	62.37	chr5:62172817-62172868	VIC	Type-it Microsatellite PCR	28	1	59	25	62374962	202,120
	end	62,172,843	5_en_62.17-R	59.35	GCGAGGGCAGAAGCAAATCT	0-5	62.37			Type-it Microsatellite PCR	28	1	59	25	62374962	202,120
Tgu6_random	centromere	566,411	6rand_cen_0.57-F	59.09	CGGGAGTCCCTTTCCTGTTT	0-2	36.31	chr6_random:566397-566425	NED	Type-it Microsatellite PCR	21	1	58	29	36305782	
	centromere	566,411	6rand_cen_0.57-R	59.09	CTTGGCAACTGGGCTGCAT	0-2	36.31			Type-it Microsatellite PCR	21	1	58	29	36305782	
Tgu6	centromere	887,848	6_cen_0.89-F	61.4	CCATGCAGCACCTCCTTCTC	0-2	36.31	chr6:887831-887865	6FAM	Type-it Microsatellite PCR	34	1	58	29	36305782	887,848
	centromere	887,848	6_cen_0.89-R	61.4	AGTGTCAGCGTGGGAGCTGT	0-2	36.31			Type-it Microsatellite PCR	34	1	58	29	36305782	887,848
Tgu6	end	35,985,882	6_en_35.99-F	59.35	CAGGGGTTGCTTTGTGCTGT	0-2	36.31	chr6:35985856-35985908	NED	Type-it Microsatellite PCR	28	1	59	25	36305782	319,900
	end	35,985,882	6_en_35.99-R	59.35	ACAGAAGCAAGGGGGGAGGAA	0-2	36.31			Type-it Microsatellite PCR	28	1	59	25	36305782	319,900
Tgu7	centromere	4,645,550	7_cen_4.65-F	60.18	GCTCCCAGTGGCTAAACCAA	4-12	39.84	chr7:4645478-4645621	VIC	Type-it Microsatellite PCR	23	0.5	59	27	39844632	
	centromere	4,645,550	7_cen_4.65-R	60.18	TGCTGCTGAATTCTGACTCACTTG	4-12	39.84			Type-it Microsatellite PCR	23	0.5	59	27	39844632	
Tgu7	end	39,175,422	7_en_39.18-F	61.4	ACTGGGGACACAGCGAGAGA	4-12	39.84	chr7:39175399-39175445	6FAM	Multiplex PCR	13	0.5	57	29	39844632	669,210
	end	39,175,422	7_en_39.18-R	61.4	CCTCGACTGGTCTGCAGCTT	4-12	39.84			Multiplex PCR	13	0.5	57	29	39844632	669,210
Tgu8	centromere	1,381,272	8_cen_1.38-F	59.585	tgGCCCAACTTGGAGACTTG	0-3	27.99	chr8:1381251-1381293	PET	Type-it Microsatellite PCR	32	0.5	58	24	27993427	1,381,272
	centromere	1,381,272	8_cen_1.38-R	59.585	TGGTATTGCTTCCCCAGCCTA	0-3	27.99			Type-it Microsatellite PCR	32	0.5	58	24	27993427	1,381,272
Tgu8	end	27,405,901	8_en_27.41-F	61.4	CAGAACCCCAGGCAGGAAAG	0-3	27.99	chr8:27405882-27405919	VIC	Type-it Microsatellite PCR	15	0.5	61	24	27993427	587,527
	end	27,405,901	8_en_27.41-R	61.4	agtgcagtgcaGGAGCCAGT	0-3	27.99			Type-it Microsatellite PCR	15	0.5	61	24	27993427	587,527
Tgu9	start	956,118	9_st_0.96-F	57.585	CCTGCAGCTTTGGCTTTTGT		27.24	chr9:956081-956155	VIC	Type-it Microsatellite PCR	20	1	57	28	27241186	956,118
	start	956,118	9_st_0.96-R	57.585	CATGCTCCCAAAGCAGTTTCA		27.24			Type-it Microsatellite PCR	20	1	57	28	27241186	956,118
Tgu9	end	26,738,326	9_en_26.74-F	60.375	CAGACCCATGACCTGCCATT		27.24	chr9:26738301-26738351	VIC	Type-it Microsatellite PCR	33	0.5	60	27	27241186	502,860
	end	26,738,326	9_en_26.74-R	60.375	GGCTGAAGCTCTGGTGCCTA		27.24			Type-it Microsatellite PCR	33	0.5	60	27	27241186	502,860
Tgu10	start	864,573	10_st_0.86-F	57.845	GGCACTTGTACATGTTTGCATC		20.81	chr10:864547-864599	6FAM	Type-it Microsatellite PCR	20	0.5	57	28	20806668	864,573
	start	864,573	10_st_0.86-R	57.845	AGCCATTCAGACCCCTTGAT		20.81			Type-it Microsatellite PCR	20	0.5	57	28	20806668	864,573
Tgu10	end	20,563,443	10_en_20.56-F	59.35	GGATTTCCTTGCCCAGCCTA		20.81	chr10:20563425-20563460	6FAM	Type-it Microsatellite PCR	28	1	59	25	20806668	243,226
	end	20,563,443	10_en_20.56-R	59.35	TGGCAGCTGTTCCATGAGTG		20.81			Type-it Microsatellite PCR	28	1	59	25	20806668	243,226
Tgu11	start	141,273	11_st_0.14-F	59.35	CAGCTGCAAAGGGCAAATCC		21.40	chr11:141254-141291	PET	Type-it Microsatellite PCR	28	1	59	25	21403021	141,273
	start	141,273	11_st_0.14-R	59.35	TTGCTGAGTGTGCCAACCAC		21.40			Type-it Microsatellite PCR	28	1	59	25	21403021	141,273
Tgu11	end	20,803,274	11_en_20.8-F	59.935	CCCCGACCGAGGTGAACT		21.40	chr11:20803254-20803294	NED	Type-it Microsatellite PCR	34	0.5	58	29	21403021	599,747
	end	20,803,274	11_en_20.8-R	59.935	GGGCTGAAATGTCCAACCTG		21.40			Type-it Microsatellite PCR	34	0.5	58	29	21403021	599,747
Tgu12	start	768,022	12_st_0.77-F	57.585	TCTTGCTTTGTCCTCCCACT		21.58	chr12:768006-768037	PET	Type-it Microsatellite PCR	20	2.5	57	28	21576510	768,022
	start	768,022	12_st_0.77-R	57.585	TGGTTATCTTGCCCTGCTTCA		21.58			Type-it Microsatellite PCR	20	2.5	57	28	21576510	768,022
Tgu12	end	20,787,953	12_en_20.79-F	60.61	GGAACTCCGGTGAGATGCTG		21.58	chr12:20787928-20787978	PET	Multiplex PCR	13	2	57	29	21576510	788,557
	end	20,787,953	12_en_20.79-R1	60.61	TGTTACAGCAGCCCAAACTGC		21.58			Multiplex PCR	13	2	57	29	21576510	788,557
Tgu13	start	365,410	13_st_0.37-F	60.165	AGGACATGGGTGGCTGGAC		16.96	chr13:365392-365428	PET	Type-it Microsatellite PCR	6	1	59	26	16962381	365,410
	start	365,410	13_st_0.37-R	60.165	CCATTGGCACTGCTCACTCA		16.96			Type-it Microsatellite PCR	6	1	59	26	16962381	365,410
Tgu13	end	16,753,185	13_en_16.75-F	59.35	CTGCTTGGTCAGCCTTGTCA		16.96	chr13:16753161-16753208	6FAM	Type-it Microsatellite PCR	22	1	59	27	16962381	209,197
	end	16,753,185	13_en_16.75-R	59.35	ATGCTGTCCAGGGCAGAGAA		16.96			Type-it Microsatellite PCR	22	1	59	27	16962381	209,197
Tgu14	start	1,074,386	14_st_1.07-F	59.35	GCCCACTCCAGAGCATTTGA		16.42	chr14:1074368-1074403	PET	Type-it Microsatellite PCR	22	1	59	27	16419078	1,074,386
	start	1,074,386	14_st_1.07-R	59.35	AAGAGGCACCTCACGCTGAA		16.42			Type-it Microsatellite PCR	22	1	59	27	16419078	1,074,386
Tgu14	end	15,435,334	14_en_15.44-F	61.78	CCAGTTGCCTCTAACCAGAGC		16.42	chr14:15435303-15435365	VIC	Type-it Microsatellite PCR	30	0.5	60	28	16419078	983,744
	end	15,435,334	14_en_15.44-R	61.78	AAGGACAAGGGAGGCTGGTTC		16.42			Type-it Microsatellite PCR	30	0.5	60	28	16419078	983,744
Tgu15	start	880,198	15_st_0.88-F	59.35	TCCTGCCATGGCTGATCACT		14.43	chr15:880176-880219	NED	Type-it Microsatellite PCR	33	1	60	27	14428146	880,198
	start	880,198	15_st_0.88-R	59.35	ATGCCAAGCAGGGACTGATG		14.43			Type-it Microsatellite PCR	33	1	60	27	14428146	880,198
Tgu15	end	13,763,436	15_en_13.76-F	59.35	GCACCAAAACCCCCTTCACT		14.43	chr15:13763410-13763462	6FAM	Type-it Microsatellite PCR	24	0.5	59	27	14428146	664,710
	end	13,763,436	15_en_13.76-R	59.35	CTGCATGACCCCTCCCTATT		14.43			Type-it Microsatellite PCR	24	0.5	59	27	14428146	664,710
Tgu17	start	702,807	17_st_0.7-F	59.35	GGGGAGGCTTGGACAGAAAT		11.65	chr17:702791-702822	6FAM	Type-it Microsatellite PCR	23	1	59	27	11648728	702,807
	start	702,807	17_st_0.7-R	59.35	GAAATCCTCAAGGGGCACAC		11.65			Type-it Microsatellite PCR	23	1	59	27	11648728	702,807
Tgu17	end	11,114,332	17_en_11.11-F	61.19	agggTGTGCAGTGCCTGTG		11.65	chr17:11114287-11114377	NED	Type-it Microsatellite PCR	15	1.5	61	24	11648728	534,396
	end	11,114,332	17_en_11.11-R	61.19	GCCCTGATGACCACTTCAGC		11.65			Type-it Microsatellite PCR	15	1.5	61	24	11648728	534,396
Tgu18	start	477,589	18_st_0.48-F	59.585	CCAGGGTGCAGTGGAACTTT		11.20	chr18:477576-477601	6FAM	Type-it Microsatellite PCR	27	0.5	59	25	11201131	477,589
	start	477,589	18_st_0.48-R	59.585	TGAGCTGAGTGCTTTCCCTGA		11.20			Type-it Microsatellite PCR	27	0.5	59	25	11201131	477,589
Tgu18	end	10,641,511	18_en_10.64-F	60.035	CGAAAGTGTTGTGTGTGGGAAG		11.20	chr18:10641492-10641529	6FAM	Type-it Microsatellite PCR	3	1	59	25	11201131	559,621
	end	10,641,511	18_en_10.64-R	60.035	ACACTGCAGCACAAAGGAAGC		11.20			Type-it Microsatellite PCR	3	1	59	25	11201131	559,621
Tgu19	start	967,796	19_st_0.97-F	61.4	TGGGCTGAGGTTCCCTCTCT		11.59	chr19:967778-967814	6FAM	Type-it Microsatellite PCR	26	0.5	60	27	11587733	967,796
	start	967,796	19_st_0.97-R	61.4	CACACCTTCAGGGCAGAAC		11.59			Type-it Microsatellite PCR	26	0.5	60	27	11587733	967,796
Tgu19	end	11,220,412	19_en_11.22-F	61.4	GGCCAGGGAAAGCAACACTG		11.59	chr19:11220288-11220535	PET	Type-it Microsatellite PCR	30	1.5	60	28	11587733	367,322
	end	11,220,412	19_en_11.22-R	61.4	GACAAGGGAGCATCCAGCAC		11.59			Type-it Microsatellite PCR	30	1.5	60	28	11587733	367,322
Tgu20	start	1,912,665	20_st_1.91-F	59.35	TGGTCGAAACTCCACGTGTC		15.65	chr20:1912652-1912678	NED	Type-it Microsatellite PCR	23	0.5	59	27	15652063	1,912,665
	start	1,912,665	20_st_1.91-R	59.35	GCCTTGGTGACTTTGCTTGC		15.65			Type-it Microsatellite PCR	23	0.5	59	27	15652063	1,912,665
Tgu20	end	15,239,517	20_en_15.24-F	59.8	TGTCAAGCAAGCTGGCAGAG		15.65	chr20:15239496-15239537	VIC	Type-it Microsatellite PCR	27	1.5	59	25	15652063	412,547
	end	15,239,517	20_en_15.24-R	59.8	GCCTCCTGGTTAAGAAAACTGC		15.65			Type-it Microsatellite PCR	27	1.5	59	25	15652063	412,547
Tgu21	start	218,485	21_st_0.22-F	59.35	CCAACAACGAGTGGGTGGAT		5.98	chr21:218465-218505	VIC	Type-it Microsatellite PCR	24	1	59	27	5979137	218,485
	start	218,485	21_st_0.22-R	59.35	GATCTCCTCACGGCATGGAA		5.98			Type-it Microsatellite PCR	24	1	59	27	5979137	218,485
Tgu21	end	5,795,144	21_en_5.8-F	59.35	GGGAATTGTGTGGTGGGATG		5.98	chr21:5795132-5795156	VIC	Type-it Microsatellite PCR	29	0.5	59	28	5979137	183,993
	end	5,795,144	21_en_5.8-R	59.35	TGCTGTTGGAAACCCTCTGG		5.98			Type-it Microsatellite PCR	29	0.5	59	28	5979137	183,993
Tgu22	start	129,952	22_st_0.13-F	59.35	TTCCTCACACCCCATCAAGC		3.37	chr22:129889-130015	NED	Type-it Microsatellite PCR	3	1	59	25	3370227	129,952
	start	129,952	22_st_0.13-R	59.35	TGTGTCCCCTCAAGCATTCC		3.37			Type-it Microsatellite PCR	3	1	59	25	3370227	129,952
Tgu22	end	2,607,525	22_en_2.61-F	59.35	CGGAGCTACGCAGGCATTTA		3.37	chr22:2607499-2607550	PET	Type-it Microsatellite PCR	25	1	59	27	3370227	762,703
	end	2,607,525	22_en_2.61-R	59.35	GCTGTGATGTGGCGAGGATT		3.37			Type-it Microsatellite PCR	25	1	59	27	3370227	762,703
Tgu22	end	3,102,107	22_en_3.1-F	59.35	CCTTGGGCCACATTCCTCTT		3.37	chr22:3102087-3102127	PET	Type-it Microsatellite PCR	23	0.5	59	27	3370227	268,120
	end	3,102,107	22_en_3.1-R	59.35	GGATTGGGCTCTGGGTGTTT		3.37			Type-it Microsatellite PCR	23	0.5	59	27	3370227	268,120
Tgu23	start	684,491	23_st_0.68-F	61.4	CTCAGGAGGCTTCTCCAGCA		6.20	chr23:684467-684515	NED	Type-it Microsatellite PCR	25	0.5	59	27	6196912	684,491
	start	684,491	23_st_0.68-R	61.4	GGAGTTGGAAGGCTGCAGTG		6.20			Type-it Microsatellite PCR	25	0.5	59	27	6196912	684,491
Tgu23	end	6,191,950	23_en_6.19-F	59.35	CCAGCTCCTCCAACATTCCA		6.20	chr23:6191935-6191964	6FAM	Type-it Microsatellite PCR	29	1	59	28	6196912	4,963
	end	6,191,950	23_en_6.19-R	59.35	ACCGGGACCATCCTGAAATC		6.20			Type-it Microsatellite PCR	29	1	59	28	6196912	4,963
Tgu24	start	409,633	24_st_0.41-F	61.4	ACCTGGGGCATCCTCTCAAG		8.02	chr24:409595-409671	PET	Type-it Microsatellite PCR	15	1	61	24	8021379	409,633
	start	409,633	24_st_0.41-R	61.4	CAGAGGAAACCTGCCAGTGC		8.02			Type-it Microsatellite PCR	15	1	61	24	8021379	409,633
Tgu24	end	7,891,710	24_en_7.89-F	57.585	AGATTTCCCCCTGCAAAACC		8.02	chr24:7891687-7891732	6FAM	Multiplex PCR	1	1	57	29	8021379	129,670
	end	7,891,710	24_en_7.89-R	57.585	TTTAACAGGGCTTTCCTCCAC		8.02			Multiplex PCR	1	1	57	29	8021379	129,670
Tgu25	start	27,765	25_st_0.03-F	59.585	GGTCGGTGATTTGGGGAGTT		1.28	chr25:27694-27836	VIC	Type-it Microsatellite PCR	22	1	59	27	1275379	27,765
	start	27,765	25_st_0.03-R	59.585	cgTCGTACTCGATGCCATGTT		1.28			Type-it Microsatellite PCR	22	1	59	27	1275379	27,765
Tgu25	end	1,224,067	25_en_1.22-F	59.35	CCTTGGGATCACCCCTTGAT		1.28	chr25:1224054-1224079	NED	Type-it Microsatellite PCR	4	1	59	26	1275379	51,313
	end	1,224,067	25_en_1.22-R	59.35	CCCGACAGGAAACATCCTCA		1.28			Type-it Microsatellite PCR	4	1	59	26	1275379	51,313
Tgu26	start	198,079	26_st_0.2-F	61.4	agacTGGGTGGGGGTGAAAG		4.91	chr26:198063-198095	PET	Type-it Microsatellite PCR	26	0.5	60	27	4907541	198,079
	start	198,079	26_st_0.2-R	61.4	CTCCTGGTCCTGATGCTTGG		4.91			Type-it Microsatellite PCR	26	0.5	60	27	4907541	198,079
Tgu26	end	4,784,177	26_en_4.78-F	61.4	GGAGCTGTGGGACTTTGTGG		4.91	chr26:4784148-4784205	6FAM	Type-it Microsatellite PCR	15	1	61	24	4907541	123,365
	end	4,784,177	26_en_4.78-R	61.4	GCTGAACCCAAGGCTCTTCC		4.91			Type-it Microsatellite PCR	15	1	61	24	4907541	123,365
Tgu27	start	575,924	27_st_0.58-F	59.35	TTGGTTTGGCCTGGACTCTG		4.62	chr27:575894-575953	PET	Type-it Microsatellite PCR	27	1	59	25	4618897	575,924
	start	575,924	27_st_0.58-R	59.35	GCCATCAAGCTGTGCCCTAA		4.62			Type-it Microsatellite PCR	27	1	59	25	4618897	575,924
Tgu27	end	4,566,154	27_en_4.57-F	59.35	GAACATCCGGAGCCCGATTA		4.62	chr27:4566130-4566177	NED	Type-it Microsatellite PCR	29	2	59	28	4618897	52,744
	end	4,566,154	27_en_4.57-R	59.35	GAGCTTCTGTCTGCCCGAAA		4.62			Type-it Microsatellite PCR	29	2	59	28	4618897	52,744
Tgu28	start	288,173	28_st_0.29-F	57.3	CTCTGCATCTTTGGCAGCAT		4.96	chr28:288144-288201	NED	Type-it Microsatellite PCR	20	0.5	57	28	4963201	288,173
	start	288,173	28_st_0.29-R	57.3	AAAGCCGTTTGGGAATCTGG		4.96			Type-it Microsatellite PCR	20	0.5	57	28	4963201	288,173
Tgu28	end	4,931,403	28_en_4.93-F	61.4	TGCCACCCTTCTACCTGCTG		4.96	chr28:4931380-4931425	NED	Type-it Microsatellite PCR	33	1	60	27	4963201	31,799
	end	4,931,403	28_en_4.93-R	61.4	TGGAGCTGTGTCCCTCCATC		4.96			Type-it Microsatellite PCR	33	1	60	27	4963201	31,799
TguZ	centromere	27,506,745	Z_cen_27.51-F	59.82	GAGCTGCAATGAGGAGCCAAA	27.6-28.1	72.86	chrZ:27506703-27506787	VIC	Type-it Microsatellite PCR	32	0.5	58	24	72861351	
	centromere	27,506,745	Z_cen_27.51-R	59.82	AACTGAGACCTATGCCCAGGA	27.6-28.1	72.86			Type-it Microsatellite PCR	32	0.5	58	24	72861351	
TguZ	end	72,806,278	Z_en_72.81-F	60.375	CCATGACCACCTGAGCCCTA	27.6-28.1	72.86	chrZ:72806256-72806300	NED	Type-it Microsatellite PCR	30	0.5	60	28	72861351	55,073
	end	72,806,278	Z_en_72.81-R	60.375	GCTTCTCGCCCTCTTTTTGC	27.6-28.1	72.86			Type-it Microsatellite PCR	30	0.5	60	28	72861351	55,073

Article title: Triploidy mapping of centromeres of microchromosomes in the zebra finch (*Taeniopygia guttata*)

Authors: Ulrich Knief and Wolfgang Forstmeier
Affiliations: Department of Behavioural Ecology and Evolutionary Genetics, Max Planck Institute for Ornithology, 82319 Seewiesen, Germany
E-mail: ukneif@orn.mpg.de
Journal: Chromosoma
