# Supplementary material for: Mapping centromeres of microchromosomes in the zebra finch (Taeniopygia guttata) using half-tetrad analysis
Source: Chromosoma. 2015 Dec 15;125(4):757–68. doi: 10.1007/s00412-015-0560-7 (PMC5023761; doi:10.1007/s00412-015-0560-7)
Supplement: Supplementary file 2 — Supplementary Table S2 (PDF 30 kb) [file 412_2015_560_MOESM2_ESM.pdf]

Supplementary Table S2: Additional information on every triploid individual used in this study. The day of embryonic death is unkown for those individuals that do not have an entry in that column but it occured before hatch. For each individual the number of markers indicating digynic and diandric origin of the triploidy is given and the number of uninformative markers in resepct to the parental origin of the third haploid chromosome set.

Individual ID	Population	Day of embryonic death	Digynic markers	Diandric markers	Digynic and diandric marker	Uniformative marker	Origin
B2011_187	Bielefeld	2	8	0	0	57	Maternal
B2013_227	Bielefeld	12	7	0	0	58	Maternal
B2013_236	Bielefeld	10	9	0	0	56	Maternal
B2011_017	Bielefeld	egg shell broke	3	0	0	62	Maternal
B2012_129	Bielefeld	2	15	0	0	50	Maternal
B2011_258a	Bielefeld	hatched, survived till age 0 days	14	0	0	51	Maternal
B2012_130	Bielefeld	1	15	0	0	50	Maternal
B2013_086	Bielefeld	11	17	0	0	48	Maternal
B2013_198	Bielefeld	12	15	0	0	50	Maternal
B2013_088	Bielefeld	3	16	0	0	49	Maternal
B2013_207	Bielefeld	0	18	0	0	47	Maternal
G12-1-1	Krakow		12	0	0	53	Maternal
G8-3-4	Krakow		12	0	0	53	Maternal
2005_118	Seewiesen	died early	13	0	0	52	Maternal
2006_550	Seewiesen		14	0	0	51	Maternal
2011_205	Seewiesen	1	11	0	0	54	Maternal
2011_308	Seewiesen	9	9	0	0	56	Maternal
2011_183	Seewiesen	10	8	0	0	57	Maternal
2006_486	Seewiesen		15	0	0	50	Maternal
2011_328	Seewiesen	3	12	0	0	53	Maternal
B2011_081	Bielefeld	1	0	7	0	58	Paternal
B2011_262a	Bielefeld	hatched, survived till age 22 days	0	9	0	56	Paternal
D739	Krakow	hatched, survived to adulthood	0	15	0	50	Paternal
K2012/13_154	Krakow	1	0	6	0	59	Paternal
9526	Seewiesen	hatched, survived to adulthood	0	14	0	51	Paternal
11450	Seewiesen	hatched, survived to adulthood	0	11	0	54	Paternal
2005_062	Seewiesen		0	2	0	63	Paternal
2005_198	Seewiesen		0	19	0	46	Paternal
2006_121	Seewiesen		0	13	0	52	Paternal
2006_390	Seewiesen	hatched, survived till age 9 days	0	15	0	50	Paternal
2007_158*	Seewiesen	2	0	15	0	50	Paternal
2008_322	Seewiesen		0	17	0	48	Paternal
2011_180	Seewiesen	11	19	5	3	38	Maternal + Paternal
2011_251	Seewiesen	2	6	6	2	51	Maternal + Paternal
K2012/13_125	Krakow	2	0	0	0	65	Unknown
2006_584	Seewiesen		4	4	0	57	Unknown
2011_289	Seewiesen	2	0	0	0	65	Unknown

* in Forstmeier & Ellegren (2010) this individual was labeled as 158_07 and accidentally assigned a maternal origin of the third chromosome set

Article title: Triploidy mapping of centromeres of microchromosomes in the zebra finch (*Taeniopygia guttata*)

Authors: Ulrich Knief and Wolfgang Forstmeier
Affiliations: Department of Behavioural Ecology and Evolutionary Genetics, Max Planck Institute for Ornithology, 82319 Seewiesen, Germany
E-mail: uknief@orn.mpg.de

Journal: Chromosoma
